# Evaluating the Diversity of Emergency Medicine Foundation (EMF) Grant Recipients in the Last Decade

**DOI:** 10.5811/westjem.2020.2.46497

**Published:** 2020-04-16

**Authors:** Richard D. Gordon, Nancy S. Kwon, Phillip D. Levy, Tracy E. Madsen, Marna Rayl Greenberg

**Affiliations:** * UTHealth McGovern, Department of Emergency Medicine, Houston, Texas; † Long Island Jewish Medical Center, Department of Emergency Medicine, New Hyde Park, New York; ‡ Wayne State University, Department of Emergency Medicine and Integrative Biosciences Center, Detroit, Michigan; § Alpert Medical School of Brown University, Deptartment of Emergency Medicine, Providence, Rhode Island; ¶ Lehigh Valley Hospital/University of South Florida Morsani College of Medicine, Department of Emergency & Hospital Medicine, Allentown, Pennsylvania

## Abstract

**Introduction:**

To study diversity of researchers and barriers to success among Emergency Medicine Foundation (EMF) grant recipients in the last 10 years.

**Methods:**

EMF grant awardees were approached to complete a brief survey, which included demographics, queries related to contributions to the literature, success in obtaining grants, and any perceived barriers they encountered.

**Results:**

Of the 342 researchers contacted by email, a total of 147 completed the survey for a response rate of 43%. The respondents were predominately mid to late career white-male-heterosexual-Christian with an average age of 44 years (range 25–69 years of age). With regards to training and education, the majority of respondents (50%) were either Associate or Professor clinical rank (8% instructor/resident/fellow and 31% Assistant). Sixty-two percent of the respondents reported perceived barriers to career advancement since completion of residency. The largest perceived barrier to success was medical specialty (26%), followed by gender (21%) and age (16%).

**Conclusion:**

Our survey of EMF grant recipients in the last 10 years shows a considerable lack of diversity. The most commonly perceived barriers to career advancement by this cohort were medical specialty, gender, and age. An opportunity exists for further definition of barriers and development of mechanisms to overcome them, with a goal of increased success for those that are underrepresented.

## INTRODUCTION

The United States (US) biomedical research workforce does not currently mirror the nation’s population demographically despite numerous attempts to increase diversity. This imbalance limits the promise of our biomedical enterprise for building knowledge and improving the nation’s health. 1 Diverse perspectives can bring improved collective understanding and problem-solving. Furthermore, groups of diverse problem solvers can outperform groups of high ability problem solvers. 2

Despite efforts to enhance diversity, challenges in broadening the research workforce remain. In prior reported data, National Institutes of Health (NIH) R01 applicants who self-identified their race as White were more likely to receive an award than Asian (−4 percentage points) or Black applicants (−13 percentage points). 3 Certain racial/ethnic groups are represented only minimally in biomedical research. Of the nations’ scientific research faculty positions—of those that were doctorate holders that were employed full time as ranked professors with federal support, 29.7% were female, 18.5% were Asian, 3% are African American, 4% are Hispanic, 0.01% are Native American, and 0.01% are Hawaiian/Pacific Islander. 4 There has been little increase in representation by sex, or racial and ethnic minority groups over the last 10 years despite them collectively being the most rapidly growing portion of the US population. 1 A disparity between sexes in academic medicine has also been described, 5 prompting the American Association of American Medical Colleges (AAMC) to publish committee recommendations to try an impact these differences. 6

It is unlikely that the emergency medicine (EM) research workforce differs significantly in this lack of equitable representation between race, ethnicity, and/or gender. For that reason, an American College of Emergency Physician (ACEP) Research Committee objective was put forth to collaborate with the American College of Osteopathic Emergency Physicians (ACOEP) and the Diversity and Inclusion ACEP task force on the topic of diversity in emergency medical research. As part of this objective, we had the goals of identifying the face of the current EM research workforce, researching the barriers that exist with regards to diversity, and supporting the growth of future leaders in emergency medicine research. To that end, we began with the Emergency Medicine Foundation (EMF). Founded in 1972, EMF is a 501(c) 3 nonprofit organization that is affiliated with the American College of Emergency Physicians (ACEP). EMF is a principal sponsor of funded research in EM, having awarded more than $16 million in grants to advance emergency medicine science and health policy. Recipients of these funds were felt to be reflective of EM investigators at various stages of their careers who have, by and large, chosen to focus on research, and who have the potential for obtaining future grant funding. Recognizing that extramural funding is a vital part of a research scholar’s success, we set out to determine the diversity in this representative subset of researchers in the EM community – specifically, the cohort of grant recipients from The Emergency Medicine Foundation (EMF) in the last 10 years.

## METHODS

A brief survey was developed by the ACEP Subcommittee on Diversity in Research with the goal of determining the demographics of EMF grant recipients over the last 10 years and identifying any perceived barriers faced by grantees. ACEP partnered with the Society for Academic Emergency Medicine’s (SAEM) Research Committee and an iterative process to edit the survey occurred. Using SurveyMonkey, the survey was piloted for content validity to a handful of grant recipients (non-EMF), and further iterations were made. The finished survey (Appendix) was sent in July 2018 with an email and an electronic link to EMF recipients who received funding in the last 10 years. A total of 371 EMF grant awardees were approached to complete the survey, which included queries related to contributions to the literature, success in obtaining grants, and any perceived barriers they encountered. The survey was voluntary and return responses were anonymous. Data were analyzed by simple descriptive statistics using frequencies and percentages using Microsoft Excel.

**Population Health Research Capsule**

What do we already know about this issue?

The United States biomedical research workforce does not currently mirror the nation’s population demographically despite numerous attempts to increase diversity.

What was the research question?

We set out to study diversity of researchers and barriers to success among Emergency Medicine Foundation (EMF) grant recipients.

What was the major finding of the study?

Our limited survey of EMF grant recipients in the last 10 years shows a considerable lack of diversity.

How does this improve population health?

These findings pose a risk to population health and optimally will be useful to inform future directions to enhance diversity of the EM research community.

## RESULTS

Of the 371 researchers who were contacted by email, 29 bounced back due to invalid email, leaving 342 surveys reaching an inbox. Of the 342 researchers, 55 completed the survey on first contact and 92 on second contact for a total of 147 responses, 43% response rate. Self-reported demographics are listed in Table 1. The respondents are predominately mid to late career white-male-heterosexual-Christian with an average age of 44 years (range 25–69 years of age).

With regards to training and education, the majority of respondents (50%) were either Associate or Professor clinical rank (8% instructor/resident/fellow and 31% Assistant). Of the respondents, 95% had either MD, MD-PhD, MD-Master’s degrees (2% DO, (<1%)% DO-PhD, (<1%)% Pa-C-PhD). 87% were board certified or board eligible, and 97% received EM residency training (4% grandfathered and 15% with additional residency training in other fields).

Responses show the EMF researchers have secured financial support from a wide spectrum of sources with university funding being the most common, after foundation funding. Of the respondents, 52% report securing Federal NIH funding and 45% report securing non-NIH Federal Funding (AHRQ, DOD, CDC). Additionally, 41% of respondents report participation in industry funded research. With regard to publications, 54% have more than 20 abstract presentations; 58% have more than 20 peer-reviewed manuscripts with 49% of respondents reporting this work is original research. Finally, 33% and 24% reported greater than 20 first and senior author publications respectively. Of note, a significant percentage of the respondents pursued additional training with 57% having completed fellowships (100% within the US), 45% of which had a research focus. Additionally, 58% of respondents have completed advanced research degrees, and 16% completed the ACEP sponsored Emergency Medicine Basic Research Skills (EMBRS) course.

With regards to the location of training and education, the majority of respondents are US citizens and received their education in the US (97% US citizen [4% naturalized] and 2% on Visa). All were College educated and 98% completed medical school in the US).

Most of the respondents’ (62%) perceived biases were barriers to achieving success throughout their careers. The largest perceived barriers to success were chosen medical specialty (26%), gender (21%), and age (16%). Of those who considered gender a barrier to career advancement, 31% were men, and 69% were women. However, 50% of the women who responded to the survey identified gender as a perceived barrier to their career advancement (vs 10% of men). The median age of those who considered age a barrier to success was 43 years. The remaining responses to perceived barriers include the following: race (7%), country of origin (2%), medical school/degree (3%), residency training site (2%), and a combination of biases (19%); 38% reported perceiving no biases.

Regarding the women who responded to the survey about the quality of their career mentorship, 80% (N=31/39) described it as either good or excellent, while 12% (N=6/39) felt it was only fair. There were only two women that felt their mentoring was poor. Comparatively, only 65% (N=58/89) of the men who responded to the survey topic rated the quality of their career mentorship good or excellent, and 27% (N=24/89) felt it was fair. Seven (8%) of the men felt the quality of their career mentorship was poor.

## DISCUSSION

At the October 2017 meeting of the ACEP Research Committee which took place in Washington DC, the challenges of developing a diverse group of EM researchers was discussed. The Committee concluded that the evidence-based literature acknowledges that a lack of diversity exists in the academic research community. An objective was set to develop a survey to better inform the EM research community about gaps in training and mentorship, with an eye towards developing interventions aimed at addressing these gaps. This survey identified several important aspects that affect diversity amongst researchers in the EM community. Based on the group that responded, a majority were of Associate Professor or Professor ranking (50%), male (67%), white (73%), US Citizens (97%) and heterosexual (87%). The majority perceived biases as barriers to success throughout their career (62%), with the largest barriers being medical specialty (26%) and gender (21%). The results of this EMF survey are not surprising and support the perception that our specialty is facing barriers to the development of a diversified research workforce, and that focused efforts need to be initiated to achieve this goal.

Some evidence suggests that creating a diverse research environment requires an integrated set of interventions. Similarly to biomedical research itself, these interventions would rely on a reasoned evidence-based approach that is rooted in the scientific methods 1 One approach would be utilization of the pipeline metaphor: “the pipeline is filled with talent waiting to be developed, and that increased emphasis must be placed on providing ongoing, active guidance rather than relatively passive provision of experiences.”^7^ Despite some evidence supporting interventions to improve diversity, other data support that intervention programs may lack effectiveness in closing this gap.^8^

Our data provide insight into the unique difficulties for certain populations to be competitive for grant funding. While women were underrepresented in our respondent sample, it is notable that nearly half (compared to just under 20% of men) found gender to be a barrier to success. The category of “other” barriers included a range of narrative responses that offer specific insight into this finding, with comments such as “being a mother,” “maternity leave,” “lack of a nuclear family,” and “backstabbing insecure leadership being provided.” These poignant reflections suggest that future research must incorporate a qualitative approach to define further what obstacles exist. The solution to diversity in funding can’t be adequately evaluated if we don’t truly understand the impediments for the full complement of academic emergency physicians.

Diversity in Emergency Medicine research should also be considered in the context of the merger between the American Osteopathic Association (AOA) and the Accreditation Council for Graduate Medical Education (ACGME). The ACEP Research Committee’s objective also included assessing the scholarly work of researchers from both training backgrounds (DO and MD). We found that only a minority of EMF grant recipients were doctors of osteopathic medicine. Studies have shown that osteopathic EM residencies are under-represented in top tier EM journal publications and very few editors of top tier academic journals are osteopathic physicians.^9^,^10^ Additionally very few osteopathic physicians have published in the *Journal of Emergency Medicine*, *Academic Emergency Medicine*, or *Annals of Emergency Medicine* over the last two decades despite a trend for increased publication by publication of allopathic physicians; notably, there was not a similar trend for increased publication of osteopathic physicians in emergency medicine. 11 A recent study aimed at determining if a medical degree disparity (between allopathic and osteopathic) existed between those who successfully received an EM R01 grant and those who did not. This study found that allopathic physicians comprised the majority of recipients who were awarded an R01 grant in EM over the last decade. Those physicians typically had numerous prior publications and an advanced degree. 12 It would seem that moving forward, discussions should also include how to encourage the development of seasoned osteopathic researchers in our EM community.

A significant limitation of this survey is the response rate of 43%, and participants may not reflect perspectives from the overall cohort. Our survey instrument was anonymous so that respondents would answer as openly as possible; unfortunately, this precluded the ability to re-send the survey to non-responders. Consequently, there was a small response rate from subgroups with more diverse representation, making it difficult to derive information for minority groups and some (e.g., Hispanics, female to male transgender) were not represented at all. Despite this limitation, our data do provide unique information that remains useful to inform future directions to enhance the diversity of the EM research community.

## CONCLUSION

Our survey of EMF grant recipients in the last 10 years shows a considerable lack of diversity. The most commonly perceived barriers to career advancement by this cohort were medical specialty, gender, and age. An opportunity exists for further definition of barriers and development of mechanisms to overcome them, with a goal of increased success for those that are underrepresented.

## Supplementary Information



## Figures and Tables

**Table 1:** Self-reported demographics from all survey participants.

Demographic	Results
Gender	
Male	92 (67%)
Female	40 (29%)
Transgender woman	1 (0.72%)
Genderqueer/Gender non-conforming	1 (0.72%)
Prefer not to answer	4 (3%)
Race/ethnicity	
White/European	101 (73%)
Asian	14 (10%)
Asian-Indian	7 (5%)
Black/African American	3 (2%)
Mixed race/ethnicity	9 (6%)
Prefer not to answer	5 (4%)
Religion	
Christian	62 (45%)
Agnostic	35 (25%)
Jewish	14 (10%)
Atheist	10 (7%)
Buddhist	3 (2%)
Hindu	2 (1%)
Muslim	1(<1%)
Baha’i	1(<1%)
Unitarian Universalism	1(<1%)
Prefer not to answer	10 (7%)
Sexual preference	
Heterosexual	120 (87%)
Bisexual	5 (4%)
Lesbian	2 (1%)
Gay	2 (1%)
Questioning	2 (1%)
Fluid	1 (<1%)
Queer	1 (<1%)
Prefer not to answer	5 (4%)
