# Supplementary material for: Evaluating the Diversity of Emergency Medicine Foundation (EMF) Grant Recipients in the Last Decade
Source: West J Emerg Med. 2020 Apr 16;21(3):595–9. doi: 10.5811/westjem.2020.2.46497 (PMC7234692; doi:10.5811/westjem.2020.2.46497)
Supplement: Supplementary file 1 [file wjem-21-595-s001.pdf]

Thank you for participating in this survey. Our society, our specialty, and our College are increasingly diverse. Last year, ACEP began a journey to increase our focus on diversity. The College seeks to remove any barriers to advancement that, perceived or not, may restrict career success in ACEP. Your answers will be used to understand better the demographics of physician-scientists in emergency medicine; in the future, this data may be used to promote diversity among emergency medicine researchers.

Because we recognize this is very private information, all responses will be anonymous. Please contact sschneider@acep.org with any questions or concerns.

* 1. Where did you receive college/university education? (if no college/university education, please select NA)

- ◯ Inside the US
- ◯ Outside the US
- ◯ Both
- ◯ Prefer not to answer
- ◯ N/A

* 2. Where did you receive your medical school education?

- ◯ Inside the US
- ◯ Outside the US
- ◯ Both
- ◯ Prefer not to answer

* 3. Please select your medical degree:

- ◯ DO
- ◯ DO-PhD
- ◯ MD
- ◯ MD-PhD
- ◯ Prefer not to answer
- ◯ Other (please specify)

* 4. Are you board certified in emergency medicine?

- Yes
- No
- Board-eligible
- Prefer not to answer

* 5. Were you grandfathered into emergency medicine (meaning no emergency medicine residency)?

- Yes
- No
- Prefer not to answer

* 6. Where did you complete your emergency medicine residency training?

- Outside the US
- Inside the US
- Both
- Prefer not to answer

* 7. Did you receive residency training in another specialty, excluding fellowship?

- Yes
- No
- Prefer not to answer

8. If you answered yes, in what other specialty(specialties) did you receive residency training?

* 9. Was this training:

- Inside the US
- Outside the US
- Prefer not to answer

* 10. Did you complete fellowship training?

- Yes
- No
- Prefer not to answer

11. If you answered yes, in what specialty(specialties) did you complete fellowship training?

* 12. Was this training:

- ◯ Inside the US
- ◯ Outside the US
- ◯ Prefer not to answer

* 13. Did you complete a research fellowship?

- Yes
- No

* 14. If you answered yes, please enter what residency training you received, including any fellowship:

* 15. Do you have an advanced research degree (eg, MS in Clinical Research or other research degree)?

- Yes
- No

* 16. If you answered yes, what advanced research degree do you have?

* 17. Did you complete the Emergency Medicine Basic Research Skills (EMBRS) workshop?

- Yes
- No

* 18. Have you ever received research funding as a principal investigator through a grant or contract?

- ◯ Yes
- ◯ No

* 19. If you answered yes, what was the source of the funding? (Check all that apply)

- [ ] Department
- [ ] University
- [ ] Foundation (eg, EMF, SAEMF)
- [ ] Federal - NIH
- [ ] Federal - non NIH (eg, AHRQ, DOD, CDC)
- [ ] Industry
- [ ] Other (please specify)

* 20. What is the approximate number of your abstracts presented at national meetings?

- <5
- 6-20
- 21-50
- 51-100
- >100

* 21. What is the approximate number of manuscripts published in peer-reviewed journals over the course of your career?

- ◯ <5
- ◯ 6-20
- ◯ 21-50
- ◯ 51-100
- ◯ >100

* 22. How many were original research manuscripts?

- ◯ <5
- ◯ 6-20
- ◯ 21-50
- ◯ 51-100
- ◯ >100

* 23. How many were review articles?

- ◯ <5
- ◯ 6-20
- ◯ 21-50
- ◯ 51-100
- ◯ >100

* 24. For how many were you first author?

- ◯ <5
- ◯ 6-20
- ◯ 21-50
- ◯ 51-100
- ◯ >100

* 25. For how many were you senior author?

- <5
- 6-20
- 21-50
- 51-100
- >100

* 26. Please select your academic rank:

- Instructor
- Assistant Professor
- Associate Professor
- Professor
- None
- Prefer not to answer
- Other (please specify)

* 27. How would you rate the quality of the career mentorship you have received?

- Poor
- Fair
- Good
- Excellent
- Prefer not to answer

* 28. Which of the following best describes your racial and/or ethnic group? (Check all that apply)

- [ ] American Indian / Alaskan Native
- [ ] Asian
- [ ] Asian-Indian
- [ ] Black / African American
- [ ] Hispanic / Latino
- [ ] Middle Eastern / North African
- [ ] Native Hawaiian / Other Pacific Islander
- [ ] White / European
- [ ] Prefer not to answer
- [ ] Other (please specify)

* 29. Do you identify as:

- ( ) Agnostic
- ( ) Atheist
- ( ) Christian
- ( ) Hindu
- ( ) Jewish
- ( ) Muslim
- ( ) Prefer not to answer
- ( ) Other (please specify)

* 30. Do you think of yourself as:

- ◯ Bisexual
- ◯ Gay
- ◯ Lesbian
- ◯ Questioning
- ◯ Straight
- ◯ Prefer not to answer
- ◯ Other (please specify)

* 31. What gender were you assigned at birth?

- ◯ Male
- ◯ Female
- ◯ Prefer not to answer
- ◯ Other (please specify)

* 32. How do you describe your gender identity today?

- ◯ Woman
- ◯ Man
- ◯ Transgender woman
- ◯ Transgender man
- ◯ Genderqueer / Gender non-conforming
- ◯ Prefer not to answer
- ◯ Other (please specify)

* 33. What is your age?

* 34. Which of the following best describes you currently?

- US citizen by birth
- Naturalized US citizen
- Permanent resident/green card holder
- Live and work in the US on a visa
- Prefer not to answer
- Other (please specify)

* 35. Since finishing residency, have you perceived barriers to career advancement due to any of the following? (check all that apply)

- Age
- Gender
- Medical specialty
- Medical school/degree
- Country of origin
- Race or ethnicity
- Religion
- Residency training site
- Sexual orientation
- None
- Unsure
- Prefer not to answer
- Other (please specify)
